# Evaluation of the accuracy of cone beam computed tomography (CBCT) in the detection of peri-implant fenestration

**DOI:** 10.1186/s12903-024-04674-z

**Published:** 2024-08-09

**Authors:** Atefeh Gholampour, Melika Mollaei, Hodis Ehsani, Fatemeh Ghobadi, Abolfazl Hosseinnataj, Mehdi Yazdani

**Affiliations:** 1 Department of Oral and Maxillofacial Radiology, Dental Research Center, Faculty of Dentistry, Mazandaran University of Medical Sciences, Sari, Iran; 2 Dental Research Center, Student Research Committee, Faculty of Dentistry, Mazandaran University of Medical Sciences, Sari, Iran; 3 Department of Periodontology, Dental Research Center, Faculty of Dentistry, Mazandaran University of Medical Sciences, Sari, Iran; 4 Department of Biostatistics, Faculty of Health, Mazandaran University of Medical Sciences, Sari, Iran

**Keywords:** Cone beam computed tomography, Dental implant, Peri-implant fenestration

## Abstract

**Background:**

Accurate assessment of the bone supporting the implant is crucial. Early detection of bone defects around the implant can prevent the loss of bone support that ultimately leads to the loss of the implant. Therefore, the purpose of this study is to check the accuracy of CBCT in detecting peri-implant fenestrations around the implant.

**Materials & methods:**

In this laboratory study, healthy beef ribs were used. The ribs were divided into three groups of 12 (control group, 1–2 mm fenestration group, and 2–3 mm fenestration group). The blocks were cut to a length of 20 mm and 36 osteotomies with dimensions of 4 × 12 mm were made by the periodontist in order to place the implant in these bone blocks. Then the titanium implant was placed in the holes and the initial scan was performed with CBCT. In the second group, fenestration-like lesions were created on the same buccal side at a distance of 10 mm from the crest with a diameter of 1–2 mm and in the third group with a diameter of 2–3 mm, and the CBCT scan was performed again with the same parameters. Two radiologists evaluated the images twice for the presence and absence of fenestration.

**Results:**

There was no statistically significant difference between direct measurements and CBCT in the fenestration group of 1–2 mm (*p* < 0.05), but there was a significant difference between direct measurements and CBCT in the fenestration group of 2–3 mm and underestimation was observed in CBCT measurements.

**Conclusion:**

The findings of this study showed that CBCT radiography has a higher accuracy in measuring the fenestration around the implant with a smaller diameter and has an acceptable diagnostic value in detecting bone loss around the implant.

## Introduction

Approximately a quarter of all radiographs obtained for medical purposes are taken by dentists, who use radiography for a variety of diagnostic purposes. Due to their low dose and cost-effectiveness, two-dimensional imaging techniques, such as intraoral and panoramic approaches, are the most frequent modalities for pathologic conditions of the jaw; nevertheless, they have limitations including the superimposition of the structures, the underestimation of bone defects, or even lack of proper diagnosis of the defects [1, 2].

The cone beam computed tomography (CBCT) technique has been utilized in dentistry for a long time and in a variety of circumstances, including surgery, implant placement, and disease diagnosis, owing to its low cost, low dose, and appropriate performance [3, 4]. Volumetric information about the teeth and bone can be obtained with CBCT during implant placement [5]. Low bone volume and density have been linked in clinical investigations to implant failure [6].

A minimum of 1 mm of bone surrounding the implant is required for successful treatment; however, unsuitable circumstances may result in lesions that alter the structure of the alveolar bone, which is a component of the periodontal tissue. Fenestration and dehiscence are two examples of these bone abnormalities. Fenestration refers to a situation where the bone on the implant is lost and the implant is covered by periosteum and gingiva. In this situation, only the coronal portion of the implant is covered by bone [7, 8].

The importance of radiography in revealing the condition of the bone surrounding the implants is remarkable. One typical two-dimensional technique is parallel periapical radiography, which has excellent spatial resolution but is unable to demonstrate the state of non-proximal surfaces of the implant. Bone loss may begin with the buccolingual surfaces of the implant since the bone in this area is not as thick [9].

CBCT is the gold standard technique for 3D evaluation in dentistry. However, due to the beam hardening artifact, the resolution of the image deteriorates in areas with high-density structures and metal objects like titanium implants, making it challenging to assess the surrounding bone [10]. Moreover, there is some uncertainty regarding the diagnostic accuracy of CBCT in identifying alveolar bone fenestration surrounding dental implants. Improved diagnostic precision makes it possible to identify problems earlier and take action to prevent the condition more rapidly [11].

Fenestration occurs when the periosteum and gingiva are the only tissues covering the root surface due to the loss of bony structures. The marginal bone remains intact in these lesions. Tooth movement, bruxism, occlusal trauma, curved roots, and labial protrusion of the teeth are among the predisposing factors for developing fenestration [12]. An investigation of 1189 teeth among the Iranian population reported the frequency of fenestration to be 5.55% [13].

Despite the high importance of diagnosing these lesions, few studies are available on the accuracy of CBCT in diagnosing these lesions in small diameter. Also, according to the knowledge of the researchers of this project, no study has compared the diagnostic accuracy of the CS9300 CBCT device (Carestream Dental LLC, Atlanta, Georgia) in different fenestration diameters. Therefore, the purpose of this study is to investigate the accuracy of CBCT in detecting bony fenestration with different diameters around the implant.

## Materials and methods

The current in vitro study obtained ethical approval from the Ethics Committee of Mazandaran University of Medical Sciences (IR.MAZUMS.REC.1402.052). In this study, fresh bovine rib bone was used due to its similarity to the alveolar bone. The sample size was calculated to be 12 ribs in each group (total number of 36 ribs) according to Saberi et al.’s investigation [10] as well as considering the first type error of 5%, the test power of 80%, and using the following formula:

$$n=\frac{{{z}_{1-\alpha/2}}^{2}*{\sigma}^{2}}{{d}^{2}}$$

**Fig. 1:**
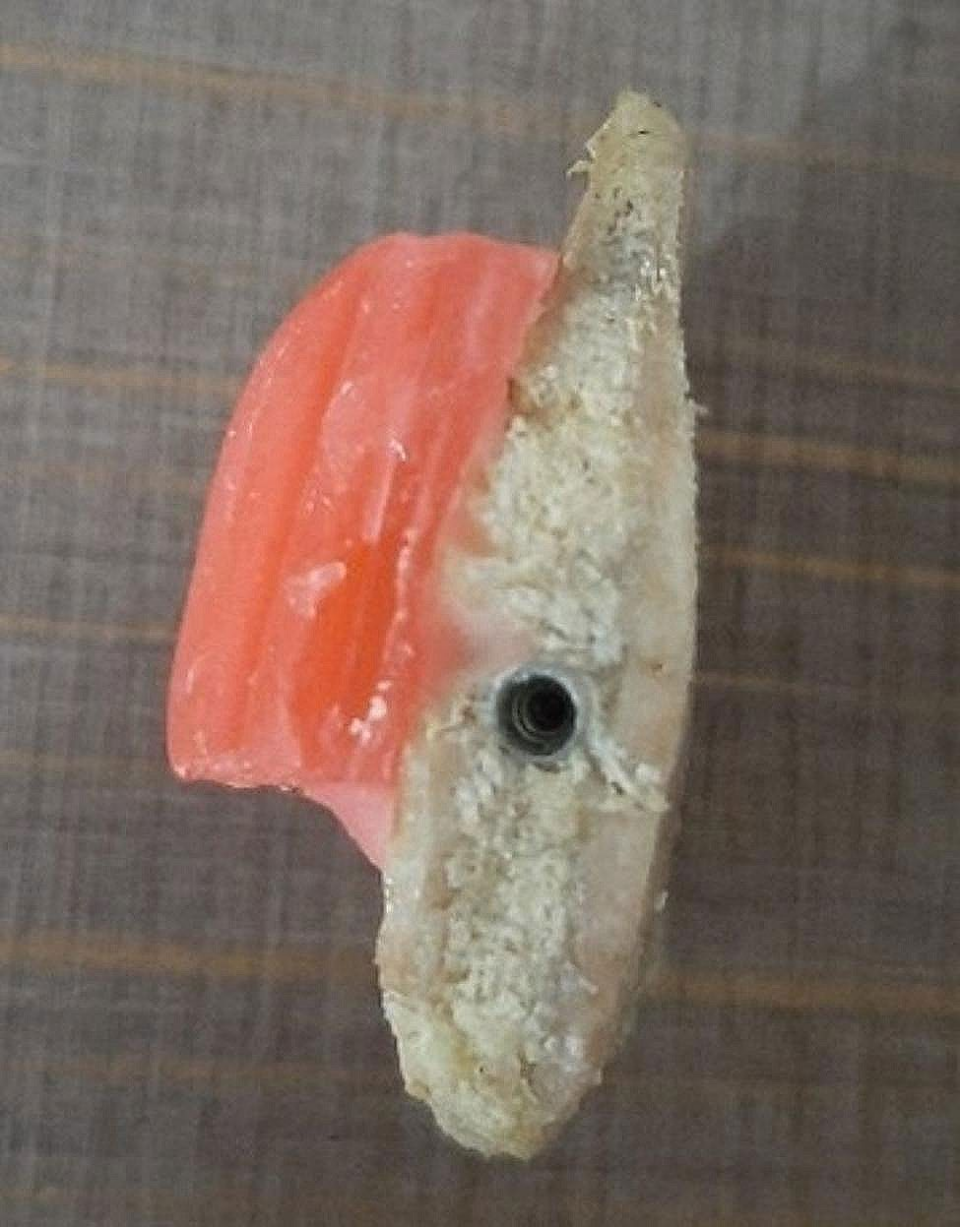
Implant osteotomy with a diameter of 4 and a length of 12 mm using the KFP dental Co implant

After removing the attached soft tissues, the ribs were cut into 20 mm long blocks. Subsequently, the implant osteotomy with a diameter of 4 mm and a length of 12 mm was performed by the periodontist (Fig. 1). Samples were randomly divided into three equal groups as follows (*n* = 12):

Group A: In this group, no fenestration was created after the osteotomy (the control group).

Group B: In this group, after implant osteotomy, fenestration was created on the same buccal side of the rib at a distance of 10 mm from the edge of the bone block using a 1–2 mm round bur.

Group C: In this group, after implant osteotomy, fenestration was created on the same buccal side of the rib at a distance of 10 mm from the edge of the bone block using a 2–3 mm round bur.

Using a digital caliper with an accuracy of 0.01 mm (Mitutoyo Series 500 − 144, Absolute, Suzano, Brazil), the fenestration diameter was monitored so that it did not exceed 2 mm in group B and 3 mm in group C.

After preparing the samples, 36 titanium implants (KFP Dental Co., Tehran, Iran) were placed in the osteotomized holes. Since the presence of soft tissue affects the contrast of CBCT images, in order to simulate the soft tissue, the ribs were covered with 1.5 cm of wax [10]. The samples were kept in the freezer between preparation and radiography in order to maintain humidity.

The samples were scanned individually by CBCT CS9300 device (Carestream Dental LLC, Atlanta, Georgia) with the following exposure parameters: field of view of 5 × 5 cm, voxel size of 90 μm, Voltage of 80 kVp, and Current 5 mA Time of 20 S.

**Fig. 2:**
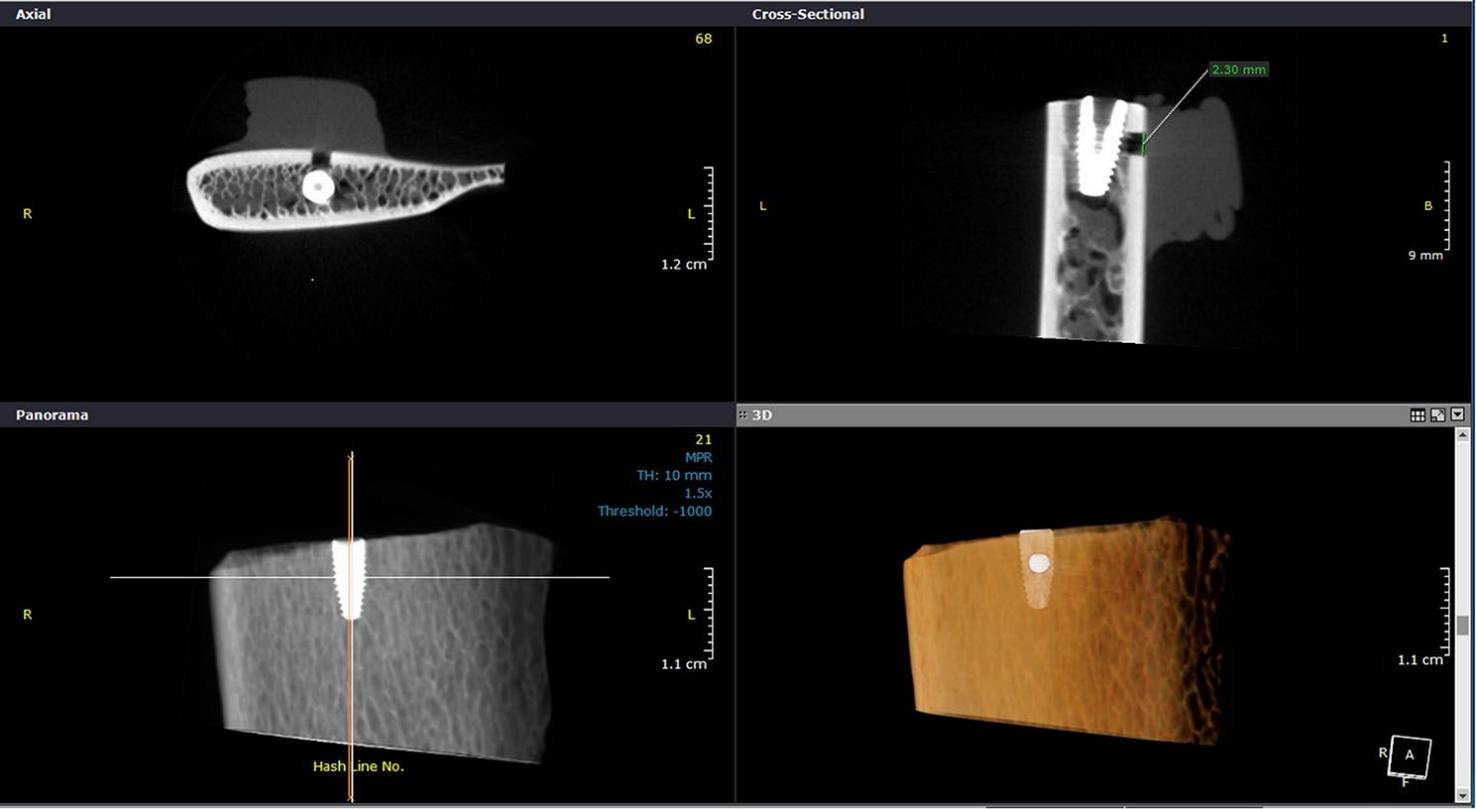
Measurement of fenestration diameter in CBCT images

The scans were reviewed separately by 2 radiologists in a semi-dark environment and an LG monitor (LCD, 20 inch, 1600 × 900 pixel). Radiologists were not aware of the presence or absence of fenestration and their diameter. Images were examined in axial, coronal, and sagittal planes and by changing the contrast, brightness, and magnification parameters for the presence or absence of bony fenestration. For each scan, the reorientation of the reconstructed images was done so that the edge of the crest was parallel to the horizon, then the panoramic view was reconstructed by drawing a curve through the center of the bone block in the axial section (Fig. 2).

Images were evaluated in axial and cross-sectional views with an interval of 0.5 mm. The maximum diameter of the fenestration (if any) was measured twice by two radiologists with a time interval of at least 1 month using the ruler tool in the OnDemand 3D Dental TM software toolbox. Finally, intra-observer reliability was calculated. The measurements obtained in direct measurement (caliper) and indirect measurements (CBCT) were compared.

In this study, the indices of sensitivity, specificity, Area under the ROC Curve (AUC), and intracluster correlation coefficient (ICC) were used to compare the diagnostic agreement. SPSS software version 22 was used for statistical calculations and the significance level was considered to be 0.05.

## Results

A total of 36 bovine ribs were divided into three groups (*n* = 12), and bone defects with a diameter of 4 mm and a length of 12 mm were created in them. The findings of evaluating the accuracy of CBCT in the diagnosis of fenestration were reported in Table 1. As observed, the accuracy of the experts’ assessment was similar and completely correct in all three groups.

**Table 1:** Evaluation of the accuracy of CBCT in fenestration diagnosis

Indicator	Group A*	Group B**	Group C***
ICC	1.0	1.0	1.0
AUC	1.0	1.0	1.0
Sensitivity	-	1.0	1.0
specificity	1.0	-	-

* Group A: No fenestration

** Group B: Fenestration with a diameter of 1–2 mm

*** Group C: Fenestration with a diameter of 2–3 mm

**Fig. 3:**
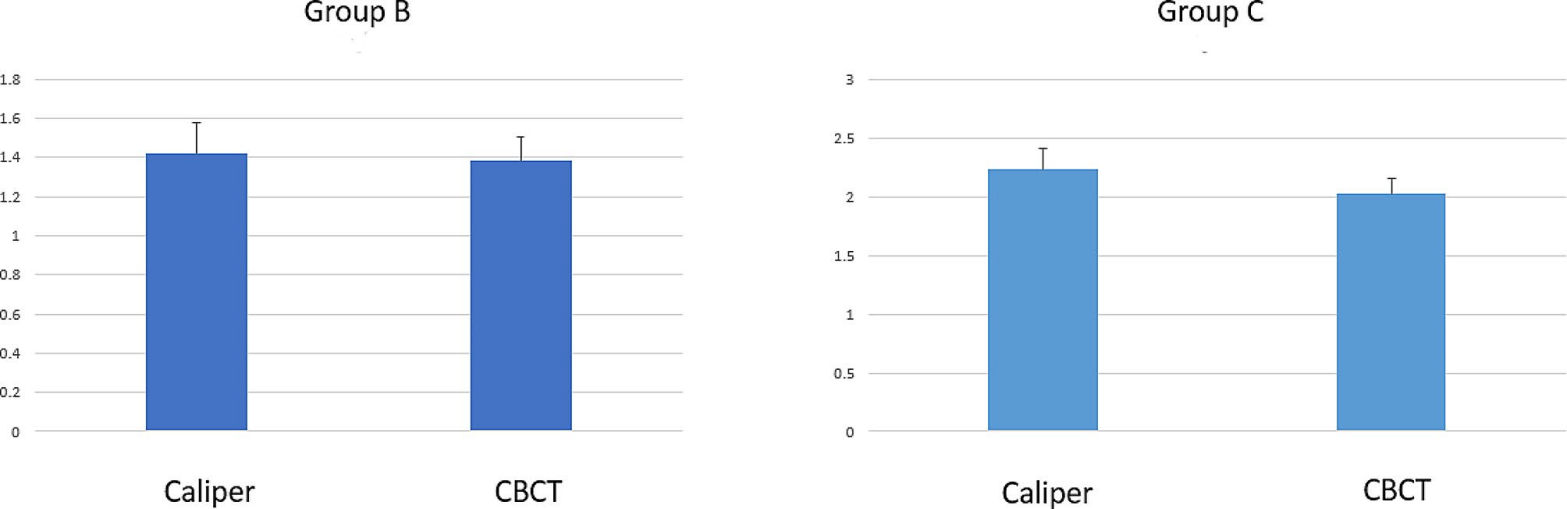
Fenestration diameter in Group B and Group C

According to the findings of Table 2, the average diameter of fenestration in group B and group C was reported and compared. There was no statistically significant difference between direct measurements and CBCT in group B (*p* < 0.05), however, there was a significant difference between direct measurements and CBCT in group C, and underestimation was observed in CBCT measurements. Considering the small mean difference (*P* = 0.541) between direct and indirect measurements in group B, it is suggested that this group had a higher measurement accuracy compared to group C (Fig. 3)

**Table 2:** Mean and standard deviation of direct measurement and CBCT scans in mm

Group	Caliper	CBCT	discrepancy		*P* value
			Mean	SD	
Group B	1/42 ± 0/16	1/38 ± 0/12	0/03	0/06	0/541
Group C	2/23 ± 0/18	2/02 ± 0/14	0/21	0/06	0/004

The ICC was used to check the agreement of the measurement methods. The ICC was 0/94 and 0/7 in group B and group C, respectively, showing acceptable agreement (Table 3).

**Table 3:** The intracluster correlation coefficient (ICC) between the groups

Group	ICC	*P* value
B	0/94	0/001<
C	0/70	0/029

## Discussion

The current study was conducted to assess the accuracy of CBCT radiography in detecting fenestration around the implant and showed that the accuracy of 100%. In addition, in the fenestration group with a smaller diameter (group B), no significant difference was observed between direct measurements and CBCT. Nevertheless, there was a significant difference between direct measurements and CBCT in the fenestration group with a larger diameter (group C), and underestimation was observed in CBCT measurements. In line with the current research, Saberi et al.‘s study also showed that CBCT radiography was 100% accurate in diagnosing angular bone defects, fenestration, and dehiscence [10]. This radiographic technique is highly accurate in diagnosing bone defects and provides acceptable results.

The CBCT device used in this study did not have metal artifact reduction (MAR) method and its effect in detecting preimplant fenestration has not been investigated. Salami et al. observed that the use of MAR does not improve the ability to detect preimplant fenestration and dehiscence [14]. On the other hand, Bagis et al. reported that the use of MAR improved the diagnostic accuracy of peri-implant fenestration [15].

In the current study, titanium implants were used. Research has suggested that in comparison to titanium or titanium alloy implants, zirconia implants create more artifacts [16, 17]. Moreover, zirconia is more likely to create artifact than titanium since it has a higher atomic number and density [18].

The current study’s results revealed that there was no statistically significant difference between group B’s direct measurements and CBCT and that CBCT has a high diagnostic value when it comes to bone loss surrounding implants. Hilgenfeld et al., Sirin et al., and Kuhl et al. demonstrated great sensitivity in detecting different peri-implant abnormalities with CBCT [12–14], which is consistent with the current investigation [9, 19, 20]. Furthermore, it has been reported by Bagis et al. and Azevedo et al. that periapical radiographs were unable to detect fenestration and dehiscence in the implant’s facial region [21, 22]. Similarly, in a Skanderlo et al. research, the detection of peri-implant fenestrations using three different CBCT systems and periapical radiography was compared. Periapical radiography was found to be ineffective in identifying defects [23].

Hence, CBCT systems were developed to address the drawbacks of traditional radiography. In this study, the diagnostic accuracy of the CBCT technique for identifying fenestration surrounding dental implants in bovine ribs was assessed. Bovine bone rib replicas are frequently employed in laboratory research Since they have cortical and cancellous bone and resemble the shape and size of the human jaw [11].

Kamburoglu et al.‘s study created dehiscence defects of various sizes in titanium implants implanted in the cadaveric mandible and classified these defects based on depth and width as small (between 1 and 3 mm), medium (between 3 and 5 mm), and large (more than 5 mm). Their findings demonstrated that compared to medium and large defects, minor ones had a poorer diagnosis accuracy [24]. Similar results were obtained in the studies of Hilgenfeld et al. and Pinheiro et al., where larger bone defects were generally measured more accurately compared to smaller ones, and there was a greater capacity to detect them as the size of the defects gradually increased [9, 25]. The findings of these studies were contrary to the results of the current study, which revealed underestimation in CBCT measurements and a significant difference between direct measurements and CBCT in the fenestration group with a greater diameter. In other words, fenestrations with a smaller diameter might be measured with more accuracy using CBCT radiography than ones with a larger diameter. The variation in the radiation dose and device type may be the cause of this discrepancy. Furthermore, Kamburoglu’s work used cadaver mandibles as samples, whereas the samples used in this investigation were bovine ribs [24].

Furthermore, varying degrees of examiner experience may also have an impact on how radiographic pictures are interpreted, according to certain research [25]. According to Pinheiro et al.‘s study, a maxillofacial surgeon with considerable training in dental implant planning was not as good at using CBCT to detect bone deformities as two oral and maxillofacial radiologists who had extensive expertise in interpreting CBCT images [26]. Utilizing the expertise of two oral and maxillofacial radiologists, the current investigation demonstrated that CBCT had a high diagnostic value for peri-implant abnormalities and that there was little variation from reference values. Moving from a laboratory to a clinical setting needs further research because of the several drawbacks that an experimental model presents.

Advantages of CBCT include images that are free of distortion and superimposition of anatomical structures. However, relatively high radiation exposure and artifacts in the vicinity of metallic objects have limited the use of this method as a routine follow-up procedure for dental implants. In contrast, periapical radiographs obtained with the parallel technique are commonly used for postoperative evaluation of implants due to their high spatial resolution and low radiation dose [10]. On the other hand, the quality of CBCT images is affected by artifacts, noise, and less contrast of soft tissue covering hard tissue. When a defect is located adjacent to a dental implant, a radiolucent area is formed. This can also occur due to beam hardening, which complicates the accurate detection of defects. Metal restorations also reduce the sensitivity of diagnostic methods. Therefore, the presence of amalgam restorations and metal veneers also reduces diagnostic accuracy [27].

Dental implants restore lost teeth to their ideal form and function while having no negative impact on the adjacent soft and hard structures. The quantity and quality of bone are critical factors in the effectiveness of dental implant treatment [23]. Improper biomechanical properties and plaque-induced inflammation are the two main factors in the development of peri-implant bone defects, which can eventually lead to the progressive loss of peri-implant bone and the loss of the implant itself if not detected [28]. Thus, radiographic examinations are crucial in order to preserve the implants as well as the surrounding bone integrity, and early detection of bone defects is highly important.

Silveiro et al. reported that peri-implant defects on the buccal side of dental implants cannot be detected on periapical radiographs, while proximal defects are easily detected [29]. A defect’s visibility on radiographs is significantly influenced by its size, shape, and location [30].

Bone defects around the implant may be created in different dimensions and shapes in a clinical situation compared to the standard forms created in the present study. Comparing fenestration in different diameters was one of the strengths of the present research. On the other hand, lack of investigating the effect of MAR on the accuracy of fenestration detection and the in vitro nature of the study was of its drawbacks. Furthermore, the bovine rib is the most similar to the alveolar bone but differs in thickness. Moreover, since soft tissue inflammation and peri-implant abnormalities coexist, the effect of such inflammation on the radiographic appearance of these defects should be evaluated in future studies.

## Conclusions

The findings of this study showed that CBCT radiography has high accuracy in detecting fenestration with different diameters around the implant. However, defects with larger diameters might be underestimated using this modality.

## Data Availability

The datasets used and/or analyzed during the current study available from the corresponding author on reasonable request.
